# `Risk of cardiovascular disease associated with febuxostat versus allopurinol use in patients with gout: a retrospective cohort study in Korea

**DOI:** 10.1007/s00296-022-05222-0

**Published:** 2022-11-08

**Authors:** Hoon Jeong, Eunmi Choi, Ahyoung Suh, Myungsik Yoo, Bonggi Kim

**Affiliations:** Office of Pharmacoepidemiology and Bigdata Analytics, Department of Drug Safety Information, Korea Institute of Drug Safety and Risk Management, 6th FL, 30, Burim-ro 169beon-gil, Dongan-gu, Anyang-si, Gyeonggi-do Republic of Korea

**Keywords:** Febuxostat, Allopurinol, Cardiovascular disease, Cohort study, Adverse drug reactions

## Abstract

Febuxostat is the drug used to treat hyperuricemia in patients with gout. Recently, the usage of Febuxostat has been controversial over the side effects in cardiovascular. The study aimed to comparatively analyze the risk of cardiovascular disease associated with febuxostat and allopurinol use in Korean patients with gout. A cohort study was conducted using national insurance claim data from the Health Insurance Review and Assessment Service (HIRA). Adult patients who were diagnosed with gout and prescribed febuxostat or allopurinol more than once from July 1, 2015, to June 30, 2018 were studied. The outcome was cardiovascular disease. Analysis was performed using Cox’s proportional hazard model following 1:1 propensity score matching to estimate the hazard ratio with a 95% confidence interval. In total, 90,590 patients were defined as the final study cohort who had an average follow-up of 467 days, including 28,732 and 61,858 patients in the febuxostat and allopurinol groups, respectively. After the 1:1 propensity score matching, the risk of cardiovascular disease in the febuxostat group was significantly higher than in the allopurinol group (HR: 1.17; 95% CI: 1.10–1.24). In the sensitivity analysis, the risk of cardiovascular disease in the febuxostat group was significantly higher than in the allopurinol group (HR: 1.09; 95% CI: 1.04–1.15). However, further sensitivity analysis showed no statistically significant difference between the febuxostat group and allopurinol group after adjusting for cardiovascular disease history before the index date. Similarly, no statistically significant difference was found between the two drugs in the subgroup analysis. Febuxostat was not associated with a significantly increased risk of cardiovascular disease.

## Introduction

Gout is a metabolic disease in which the concentration of uric acid in the blood increases, leading to the deposition of uric acid crystals in tissues, such as joints and tendons, causing inflammation and pain. Its prevalence in Korea has increased steadily from 0.17% in 2001 to 0.4% in 2008 and 2.0% in 2015 according to the National Health Insurance Service [1]. Febuxostat is the primary drug used to treat hyperuricemia in patients with chronic gout. Febuxostat is an inhibitor of xanthine oxidase, like allopurinol, but its chemical structure differs from that of allopurinol, and hence, has no cross-reactions. Febuxostat lowers uric acid levels by selectively blocking xanthine oxidase [2]. Febuxostat was covered by national insurance as second-line therapy for cases where the efficacy of allopurinol was insufficient or there was a risk of hypersensitivity in Korea. However, since July 1, 2016, these insurance benefits were expanded to include febuxostat as first-line therapy [3].

The safety of febuxostat concerning cardiovascular disease has been a controversial issue. According to the results of phase 3 clinical trials such as FACT (2005) and APEX (2008) conducted before FDA approval, febuxostat had a higher rate of cardiovascular disease death and severe cardiovascular disease than allopurinol. However, there was no physiological mechanism to explain this, and the number of cases were small; so, it was necessary to confirm the relevance through additional clinical trials [4, 5]. In the subsequent F-153 (2008) clinical trial that added the proportion of patients with cardiovascular risk factors, it was found that febuxostat did not have more cardiovascular side effects than allopurinol. However, considering that the results were different from previous clinical trials, it received conditional approval (2009) with the risk of developing cardiovascular disease to be confirmed in a post-marketing assessment [6]. After approval by FDA, in the Cardiovascular Safety of Febuxostat and Allopurinol in Patients with Gout and Cardiovascular Morbidities (CARES) trial, the risks of cardiovascular-related and all-cause mortality were found to be 1.34 and 1.22 times higher with febuxostat than with allopurinol [7]. Thus, the US Food and Drug Administration (FDA) mandated that the gout drug febuxostat carries a boxed warning to alert clinicians and patients of the increased risk of cardiovascular death with the drug in February 2019 [8]. Furthermore, the American College of Rheumatology recommended allopurinol as the first-line therapy for gout treatment in the gout management guidelines in June 2020 [9]. On the other hand, the Ministry of Food and Drug Safety in Korea recommends the use of allopurinol for patients who do not have the HLA-B*5801 gene, as confirmed by genetic testing. Allopurinol has a risk of Severe Cutaneous Adverse Drug Reactions (SCAR) side effects when the HLA-B*5801 gene is present, and the retention rate of HLA-B*5801 gene in Koreans is 12.2%, which is higher than that of Westerners 1 ~ 2% [10].

Preclinical cardiovascular studies of febuxostat had shown no toxic effects related to cardiac rhythm and function [11–15], and to date, the mechanism underlying the risk of cardiovascular death is unclear [7]. Therefore, additional studies are required to determine causalities. In this study, febuxostat was compared with allopurinol in terms of association with cardiovascular disease in gout patients to provide evidence of drug safety in Korea.

## Methods

### Data source

This study used national insurance claim data from the Health Insurance Review and Assessment Service (HIRA) in Korea. The data includes about 98% of the entire nation’s population due to the universal coverage system in Korea (97% health insurance and 3% medical aid). HIRA data contain patient information related to healthcare services such as socio-demographics, diagnoses, treatments, procedures, pharmaceuticals, medical utilization and in-hospital deaths. More than 99% of the HIRA data were collected using an electronic data interchange system and contained about 120 information lists [16].

### Study cohorts

**Fig. 1:**
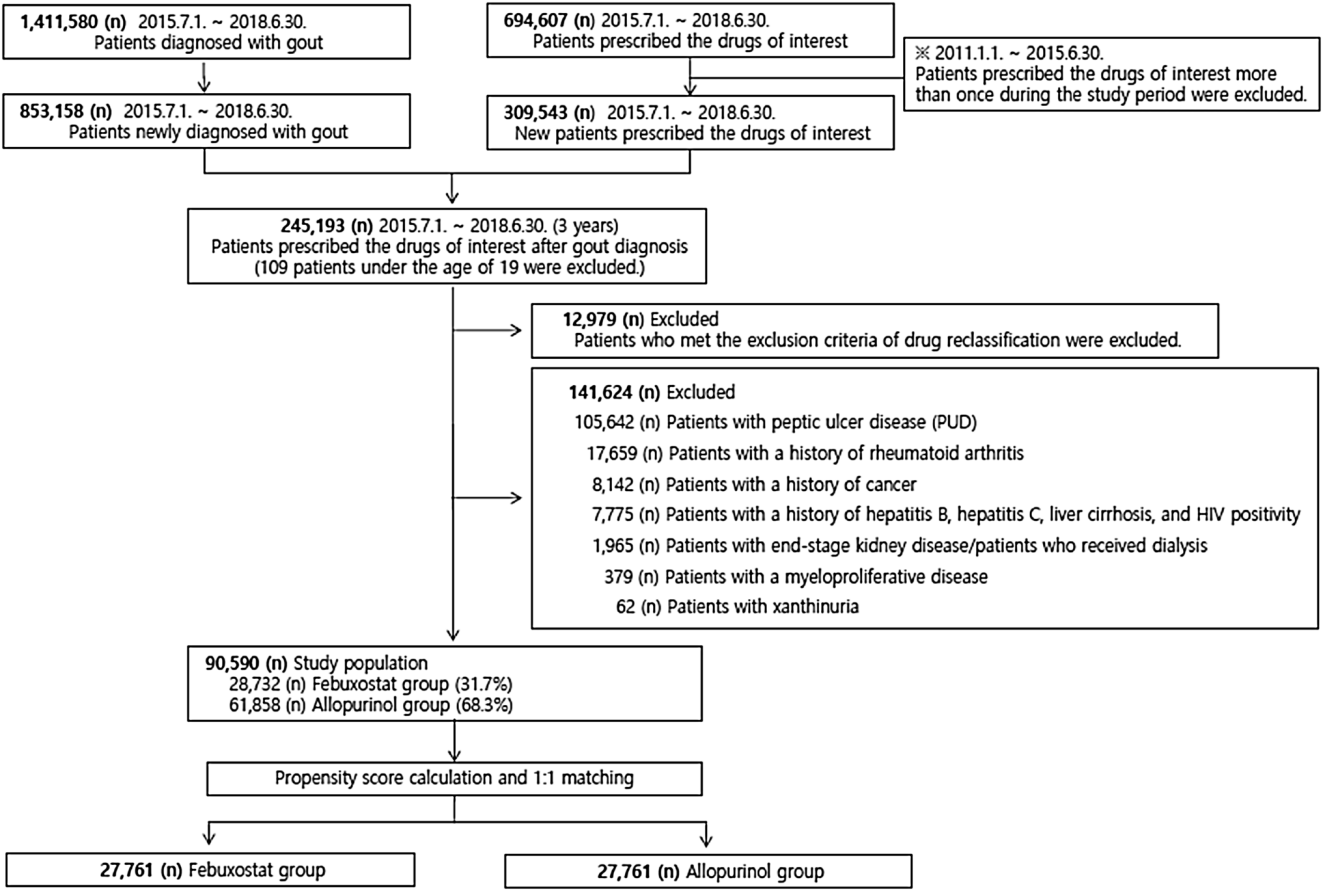
The study cohort selection process

A retrospective cohort study was performed using HIRA data from January 1, 2011, to June 30, 2018. Patients aged 19 years or older who were diagnosed with gout at least once and prescribed the study drugs (febuxostat or allopurinol) more than once from July 1, 2015, to June 30, 2018 (3 years) were selected. We identified the index date by the first prescription date of febuxostat or allopurinol for each patient and classified them into two groups (febuxostat exposed group or allopurinol control group). We excluded patients who were prescribed the study drugs (febuxostat or allopurinol) from January 1, 2011, to June 30, 2015. We also excluded patients younger than 19 years and those with a history of end-stage kidney disease/dialysis, myeloproliferative disease, xanthinuria, peptic ulcer, cancer, rheumatoid arthritis, hepatitis B, hepatitis C, liver cirrhosis, and HIV positivity before the index date (Fig. 1).

### Exposure and outcomes

**Fig. 2:**
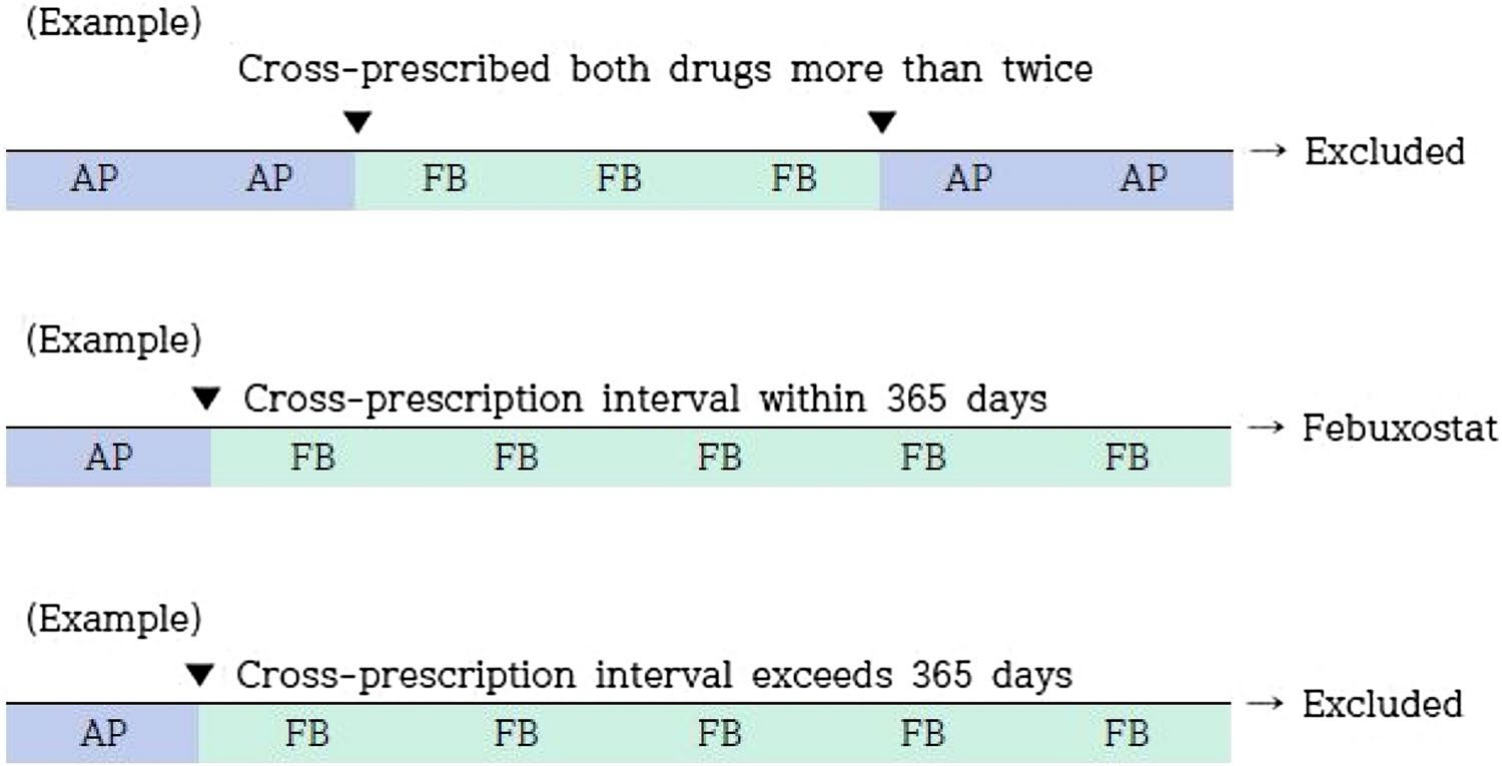
Re-classified criteria of patients who were prescribed both the drugs

We included patients who were prescribed the drugs of interest more than once after gout diagnosis. Since July 1, 2016, the insurance benefits for febuxostat were expanded in Korea; thus, we re-classified patients who were prescribed both drugs. We excluded patients who were cross-prescribed both drugs more than twice. Those who were cross-prescribed both drugs once were included in the latest drug use group if the second drug was prescribed less than 365 days after the first drug. We excluded patients who were prescribed the second drug more than 365 days after the first drug because various other confounding factors could have affected the cross-prescription of the study drugs. For this definition of exposure, we referred to a previous study (Fig. 2) [17].

The primary outcome of the study was defined as the first diagnosis of a cardiovascular disease, such as myocardial infarction, ischemic heart disease, stroke, transient ischemic attack, heart failure, coronary revascularization, and all-cause death observed during the follow-up period. Secondary outcomes were individual components of the primary outcome. Additionally, when the outcomes were observed multiple times in one patient, only the first case was included in the analysis (Appendix Table 9).

### Covariates

The confounding variables were as follows: sex, age, medication history for 1 year before the index date (such as angiotensin-converting enzyme inhibitors, calcium channel blockers, angiotensin receptor blockers, diuretics, hypoglycemic agents, anti-hyperlipidemic drugs, systemic steroids, probenecid, colchicine, and nonsteroidal anti-inflammatory drugs [NSAIDs]), disease history (such as renal failure, kidney stones, angina, diabetes, peripheral artery disease, degenerative arthritis, hypertension, hyperlipidemia, obesity, smoking, and Charlson comorbidity index [CCI]), and cardiovascular history before the index date (myocardial infarction, ischemic heart disease, stroke, transient ischemic attack, heart failure, and coronary revascularization).

### Statistical analysis

All statistical analyses were performed using the SAS Enterprise Guide 6.1 software (SAS Institute Inc., Cary, NC, United States), and significance was set at *P* < 0.05. To compare the differences in baseline characteristics between the febuxostat and allopurinol users, we examined the frequencies and percentages of the categorical variables and compared them using the chi-square test, and in some cases, Fisher’s exact test. Next, we calculated the means and standard deviations (SD) for the continuous variables. Then, we used propensity score (PS) matching (caliper 1:1 matching) to reduce the potential selection bias in observational studies and balance the distribution between the two groups by excluding confounding variables [18]. We used the Cox proportional hazard regression and estimated the hazard ratio (HR) with a 95% confidence interval (CI) for the risk of cardiovascular disease associated with febuxostat and allopurinol. As a sensitivity analysis, we limited the study period (from July 1, 2016, to June 30, 2018) to consider the time when febuxostat insurance benefit was expanded as the first-line therapy for gout in Korea and analyzed the risk of cardiovascular disease with febuxostat group compared to that with allopurinol group. We also compared the risk of cardiovascular disease between the two drug groups according to cardiovascular disease history, using the same method of PS matching (Appendix Tables 4, 5, 6, 7).

## Results

### Patient characteristics

In total, 90,590 patients were defined in the final study cohort, including 28,732 (31.7%) and 61,858 (68.3%) patients in the febuxostat and allopurinol groups, respectively. This baseline cohort comprised 90.7% males, and the mean age of the febuxostat group was 46.6 years, which was higher than that of the allopurinol group (46.3 years). Patients in the febuxostat group had a higher prevalence of renal failure, diabetes, hypertension, and hyperlipidemia compared to the allopurinol group. After 1:1 PS matching, 27,761 patients were grouped in the febuxostat group and 27,761 in the allopurinol group. All baseline covariates (medication history, CCI, and disease history) including the cardiovascular risk factors were well balanced with a standardized difference in the prevalence of < 0.1 after PS matching (Table 1).

**Table 1:** Baseline characteristics before and after propensity score matching in the febuxostat and allopurinol groups between 2015 and 2018

Classification		Before propensity score matching					After propensity score matching				
		Febuxostat (28,732 patients, 31.7%)		Allopurinol (61,858 patients, 68.3%)		STD (%) 0.2	Febuxostat (27,761 patients, 50.0%)		Allopurinol (27,761 patients, 50.0%)		STD (%) − 2.9
		*N*	(%)	*N*	(%)		*N*	(%)	*N*	(%)	
Sex	Male	26,067	90.7	56,086	90.7		25,306	91.2	25,532	92.0	
Age	Mean (standard deviation)	46.6 (± 15.6)		46.3 (± 15.2)		1.9	46.2 (± 15.4)		45.7 (± 15.1)		3.3
Medication history	Nonsteroidal anti-inflammatory drugs (NSAIDs)	21,989	76.5	47,086	76.1	1.0	21,345	76.9	21,621	77.9	− 2.4
	Systemic corticosteroids	17,152	59.7	35,352	57.2	5.2	16,665	60.0	17,058	61.5	− 2.9
	Gout medication	13,974	48.6	30,846	49.9	− 2.5	13,785	49.7	14,193	51.1	− 2.9
	Anti-hyperlipidemic agents	7,513	26.2	12,128	19.6	15.6	6,849	24.7	6,605	23.8	2.1
	Hypoglycemic agents	2,661	9.3	4,376	7.1	8.0	2,332	8.4	2,166	7.8	2.2
	Hypotensors	8,774	30.5	15,375	24.9	12.7	8,014	28.9	7,598	27.4	3.3
CCI	Mean (standard deviation)	1.0 (± 1.3)		0.8 (± 1.1)		16.6	0.9 (± 1.2)		0.9 (± 1.2)		0.0
	0	13,660	47.5	33,870	54.8	− 14.5	13,660	49.2	14,002	50.4	− 2.5
	1	7,339	25.5	16,091	26.0	− 1.1	7,335	26.4	7,415	26.7	− 0.7
	2	3,480	12.1	6,440	10.4	5.4	3,297	11.9	3,110	11.2	2.1
	3	1,721	6.0	2,671	4.3	7.6	1,501	5.4	1,431	5.2	1.1
	> 3	2,532	8.8	2,786	4.5	17.3	1,968	7.1	1,803	6.5	2.4
Disease history	Renal failure	2,779	9.7	1,851	3.0	27.7	1,829	6.6	1,796	6.5	0.5
	Kidney stone	267	0.9	437	0.7	2.5	241	0.9	227	0.8	0.6
	Angina	1,446	5.0	2,391	3.9	5.7	1,269	4.6	1,114	4.0	2.8
	Diabetes mellitus	5,073	17.7	8,224	13.3	12.1	4,553	16.4	4,251	15.3	3.0
	Peripheral arterial disease	16	0.1	11	0.0	2.0	15	0.1	4	0.0	2.1
	Degenerative arthritis	3,130	10.9	5,380	8.7	7.4	3,033	10.9	3,049	11.0	− 0.2
	Hypertension	10,402	36.2	17,725	28.7	16.2	9,525	34.3	9,063	32.7	3.5
	Hyperlipidemia	11,330	39.4	19,012	30.7	18.3	10,506	37.8	10,305	37.1	1.5
	Obesity	63	0.2	78	0.1	2.2	59	0.2	40	0.1	1.6
	Smoking	22	0.1	43	0.1	0.3	19	0.1	21	0.1	− 0.3
History of cardiovascular disease	Myocardial infarction	199	0.7	320	0.5	2.3	160	0.6	160	0.6	0.0
	Ischemic heart disease	605	2.1	908	1.5	4.8	528	1.9	460	1.7	1.9
	Stroke	698	2.4	1,226	2.0	3.0	616	2.2	557	2.0	1.5
	Transient ischemic attack	139	0.5	298	0.5	0.0	132	0.5	105	0.4	1.5
	Heart failure	1,117	3.9	1,333	2.2	10.1	942	3.4	860	3.1	1.7
	Coronary revascularization	265	0.9	410	0.7	2.9	224	0.8	191	0.7	1.4

Medication history, CCI, disease history: results of use or diagnosis for 1 year before the index date

If the absolute value of the standardized difference (STD) was higher than 10, it was judged to be different between the two groups

Propensity score matching: propensity scores of the febuxostat and allopurinol groups were matched 1:1 with a caliper width of 0.005

Average follow-up of 467 days in the febuxostat and allopurinol groups between 2015 and 2018

### Risk of cardiovascular disease associated with febuxostat and allopurinol groups

After PS matching, the risk of cardiovascular disease in the febuxostat group was significantly higher than that in the allopurinol group (HR: 1.17; 95% CI: 1.10–1.24). In the secondary outcome analysis, the risks of ischemic heart disease, stroke, and heart failure in the febuxostat group were significantly higher than those in the allopurinol group; ischemic heart disease: HR 1.19, 95% CI 1.05–1.35; stroke: HR 1.12, 95% CI 1.01–1.25, and heart failure: HR 1.16, 95% CI 1.06–1.27, respectively (Table 2).

**Table 2:** Risk of cardiovascular disease in the two drug groups after propensity score matching between 2015 and 2018

Classification	Febuxostat (27,761 patients)			Allopurinol (27,761 patients)			Crude HR (95% CI)	Adjusted HR** (95% CI)
	Number of patients (*n*)	Person–years	Incidence rate (1000 person-years)	Number of patients (*n*)	Person–years	Incidence rate (1,000 person-years)		
Primary outcome variable*								
Cardiovascular disease	2062	24,369.8	84.6	2203	39,861.9	55.3	1.17 (1.10–1.24)	1.11 (1.05–1.18)
*Secondary outcome variable*								
Myocardial infarction	139	26,150.7	5.3	171	42,528.4	4.0	1.12 (0.90–1.41)	1.05 (0.83–1.32)
Ischemic heart disease	490	25,821.1	19.0	535	42,048.6	12.7	1.19 (1.05–1.35)	1.11 (0.98–1.25)
Stroke	656	25,671.7	25.6	716	41,756.2	17.1	1.12 (1.01–1.25)	1.03 (0.93–1.15)
Transient ischemic attack	133	26,155.7	5.1	164	42,531.1	3.9	1.12 (0.89–1.41)	1.05 (0.83–1.33)
Heart failure	939	25,401.6	37.0	1,033	41,496.4	24.9	1.16 (1.06–1.27)	1.08 (0.99–1.18)
Coronary revascularization (treatment)	59	26,216.6	2.3	88	42,632.9	2.1	1.01 (0.70–1.44)	0.95 (0.66–1.36)
All-cause death	5	24,369.8	0.2	9	39,861.9	0.2	1.02 (0.33–3.14)	0.99 (0.32–3.07)

*All-cause death: includes 5 and 9 patients in the febuxostat and allopurinol groups, respectively

Coronary revascularization is a treatment code that has secondary outcome variables and overlapping values

**Covariate correction: the history of cardiovascular disease before the index date

Average follow-up of 321 and 524 days in the febuxostat and allopurinol groups, respectively

### Sensitivity analysis

In total, 68,317 patients (29,412 and 38,905 patients in the febuxostat and allopurinol groups, respectively) received febuxostat and allopurinol between 2016 and 2018. After 1:1 PS matching, 26,962 patients in the febuxostat group and 26,962 in the allopurinol group were identified. After PS matching, the risk of cardiovascular disease in the febuxostat group was significantly higher than that in the allopurinol group (HR: 1.09; 95% CI: 1.04–1.15). However, after adjusting for cardiovascular disease history before the index date, the risk of cardiovascular disease was not different between the two groups (HR: 1.04; 95% CI: 0.99–1.10) (Table 3).

**Table 3:** Risk of cardiovascular disease in the two drug groups after propensity score matching between 2016 and 2018

Classification	Febuxostat (26,962 patients)			Allopurinol (26,962 patients)			Crude HR (95% CI)	Adjusted HR** (95% CI)
	Number of patients (*n*)	Person–years	Incidence rate (1,000 person–years)	Number of patients (*n*)	Person–years	Incidence rate (1,000 person–years)		
Primary outcome variable*								
Cardiovascular disease	3127	20,343.7	153.7	3,039	24,108.4	126.1	1.09 (1.04–1.15)	1.04 (0.99–1.10)
*Secondary outcome variable*								
Myocardial infarction	197	22,689.9	8.7	219	26,634.7	8.2	0.98 (0.81–1.18)	0.88 (0.71–1.08)
Ischemic heart disease	727	22,251.4	32.7	745	26,190.9	28.4	1.05 (0.95–1.17)	0.98 (0.88–1.10)
Stroke	1100	21,951.6	50.1	1,098	25,811.0	42.5	1.06 (0.97–1.15)	1.01 (0.92–1.10)
Transient ischemic attack	235	22,657.8	10.4	219	26,630.7	8.2	1.16 (0.97–1.40)	1.10 (0.90–1.35)
Heart failure	1303	21,839.4	59.7	1,315	25,698.2	51.2	1.07 (0.99–1.15)	0.99 (0.91–1.08)
Coronary revascularization (treatment)	70	22,792.1	3.1	73	26,766.1	2.7	1.09 (0.76–1.56)	0.98 (0.64–1.49)
All-cause death	7	20,343.7	0.3	8	24,108.4	0.3	1.07 (0.39–2.96)	1.38 (0.37–5.16)

*All-cause death: includes 7 and 8 patients in the febuxostat and allopurinol groups, respectively

Coronary revascularization is a treatment code that has secondary outcome variables and overlapping values

**Covariate correction: the history of cardiovascular disease before the index date

Average follow-up of 276 and 327 days in the febuxostat and allopurinol groups, respectively

### Subgroup analysis

In the subgroup analysis considering those with and without a cardiovascular disease history before the index date, there was no statistically significant difference in the risk of cardiovascular disease between the febuxostat and allopurinol groups (Appendix Tables 4, 5, 6, 7). For the primary outcome, cardiovascular disease, the HR for the febuxostat group compared to the allopurinol group were 1.02 (95% CI: 0.95–1.10) in those with a cardiovascular disease history and 1.11 (95% CI: 0.99–1.24) in those without a cardiovascular disease history. The sensitivity analysis results were also similar between the two groups.

## Discussion

Based on our results, the risk of cardiovascular disease in the febuxostat group was significantly higher than that in the allopurinol group. However, in the sensitivity analysis, after adjusting for cardiovascular disease history before the index date, there was no statistically significant difference in the risk of cardiovascular disease between the febuxostat and allopurinol groups. Besides, in the subgroup analysis including those with and without a cardiovascular disease history, there was no statistically significant difference. From the combined results of the analyses, we concluded that febuxostat was not associated with a higher risk of cardiovascular disease than allopurinol. In addition, we have also performed subgroup analysis by sex and age. The subgroup analysis result showed a trend similar to the main analysis result. In our study, the rates of medication history, CCI, disease history, and cardiovascular disease history were significantly higher in the febuxostat group than in the allopurinol group from 2015 to 2018. In contrast, the differences in the rates between the two groups decreased after the expansion of the insurance benefits to febuxostat used as the first-line therapy. The difference in patient characteristics and drug use patterns according to the expansion of the insurance benefits may have affected the results (Appendix Table 8). Moreover, no statistically significant difference was observed between the two groups in the secondary outcomes after PS matching and adjusted for the cardiovascular disease history. Thus, there was no association between the incidence of cardiovascular disease and febuxostat.

According to a study by Zhang et al. [17], there was no statistically significant difference in the association of hospitalization for myocardial infarction or stroke between the febuxostat and allopurinol groups. Besides, the subgroup analysis stratifying those with and without baseline cardiovascular disease (including myocardial infarction, stroke, coronary revascularization, and all-cause death) showed no statistically significant difference between the febuxostat and allopurinol groups. This finding was consistent with that of our study. Moreover, in a retrospective cohort study by Kang et al. [19], the risk of cardiovascular disease (including myocardial infarction, stroke, transient ischemic attack, and coronary revascularization) was not different between the two groups. Kang et al. [19] used claim data from 2002 to 2015 in Korea, and only analyzed the period in which febuxostat was covered by insurance as a second-line therapy. However, our study used recent claim data (July 1, 2015, to June 30, 2018) including data from the period in which the insurance coverage benefit was expanded to febuxostat as first-line therapy in Korea (July 1, 2016); we analyzed the data using sensitivity analysis. In other words, this study observed the effects of febuxostat and allopurinol on the risk of cardiovascular disease under a condition in which both drugs were equally considered as first-line therapy in clinical settings. A recent meta-analysis study found that febuxostat use was not associated with an increased risk of cardiovascular events and death, all-cause mortalities [20, 21]. In addition, febuxostat was found not to be associated with an increased risk of death or serious adverse events compared to allopurinol in a recent FAST (2020) study [22]. Nevertheless, in the CARES trial [7], the risks of cardiovascular-related and all-cause mortality were 1.34 and 1.22 times higher with febuxostat than with allopurinol. The CARES trial is a randomly assigned clinical trial, which was conducted using a study method that minimized selection bias by ensuring that the study cohort had an equal probability of being assigned to each group and by normalizing all factors other than the drugs administered. However, 56.6% of patients discontinued the trial regimen prematurely, and 45.0% of patients discontinued follow-up, making it difficult to judge if the selection bias was minimized [23]. Moreover, in the CARES trial, the risk of cardiovascular-related mortality was different when a subgroup analysis was performed including the combination of the study drugs with low-dose aspirin or NSAIDs. In this study, PS matching was performed including aspirin and NSAIDs, as well as diseases and medication use that may affect cardiovascular diseases and hyperuricemia risk, thereby, minimizing potential selection bias. However, the rates of medication history (anti-hyperlipidemic agents, hypoglycemic agents, and hypotensors) and history of diseases such as kidney failure, angina, diabetes, hypertension, hyperlipidemia, and cardiovascular disease, especially heart failure, were higher in the febuxostat group than in the allopurinol group. These characteristics may have affected the risk for cardiovascular disease [24]. Furthermore, the incidences of ischemic heart disease and heart failure were higher in the febuxostat group than in the allopurinol group. Therefore, febuxostat use in patients with related conditions would need to be carefully monitored.

The key strength of this study is that it represents a large population using national claims data. The HIRA data includes about 98% of the entire nation’s population due to the universal coverage system of Korea (97% health insurance and 3% medical aid). Moreover, we included all patients aged 19 years and above in the study cohort, and the results of this study are applicable to all adult patients with gout. In particular, the results of this study are meaningful in that they differ from previous studies that analyzed the risk of cardiovascular disease associated with febuxostat in specific age groups. We also sought to minimize the effect of confounding factors, such as underlying diseases and medication use, which are known to be related to cardiovascular disease and hyperuricemia risk, by applying statistical methods such as PS matching.

Despite these strengths, this study has some limitations. First, as is inherent to any observational study, socioeconomic and clinical confounding factors that were unmeasured or unpredicted may have affected the results. Therefore, clinical characteristics such as cardiovascular complications, disease severity, and serum uric acid levels should be considered comprehensively when using febuxostat in patients with gout. Second, we obtained the maximum study period considering the approval date of febuxostat (June 2009) and the date of expansion of insurance coverage to febuxostat as first-line therapy (July 1, 2016). However, it was insufficient to assess the association between cardiovascular disease and long-term febuxostat use. Therefore, the long-term safety of febuxostat and follow-up studies should be continuously monitored. Third, we compared the risk of cardiovascular disease associated with febuxostat to that related to allopurinol and therefore, cannot conclude on the absolute impact of febuxostat use on the risk of cardiovascular disease. Therefore, it would be unreasonable to consider the risk of cardiovascular disease as a decisive factor in choosing between febuxostat and allopurinol. Finally, the association between febuxostat and cardiovascular death, which has been a controversial safety issue in other countries, could not be defined because of the nature of the insurance claim data.

In conclusion, there was a significant difference in the risk of cardiovascular disease between gout patients who were treated with febuxostat and those with allopurinol. However, after adjusting for the history of cardiovascular disease, and considering the period during which the insurance benefits of febuxostat were expanded to its use as a first-line therapy (July 1, 2016), a causal link between febuxostat and febuxostat-associated cardiovascular disease events was not established. However, the FDA has restricted the use of febuxostat to those patients who experience side effects and show no treatment effects of allopurinol, based on the results of the CARES trial, and the American College of Rheumatology has recommended allopurinol as the first-line therapy for gout treatment in the gout management guidelines in June 2020. In addition, in Korea, instructions specify that febuxostat should be carefully administered in patients with ischemic heart disease or congestive heart failure. Therefore, long-term follow-up is required when febuxostat is prescribed for patients with cardiovascular disease.

## Appendix

See Tables 4, 5, 6, 7, 8, 9, 10.

**Table 4:** Risk of cardiovascular diseases in the two drug groups after matching between 2015 and 2018 (with cardiovascular disease history)

Classification	Febuxostat (2763 patients)			Allopurinol (2763 patients)			HR (95% CI)
	Number of patients (*n*)	Person–years	Incidence rate (1000 person –years)	Number of patients (*n*)	Person–years	Incidence rate (1000 person –years)	
*Primary outcome variable**							
Cardiovascular disease	1464	1401.9	1044.3	1579	2034.6	776.1	1.02 (0.95–1.10)
*Secondary outcome variable*							
Myocardial infarction	89	2718.2	32.7	108	4,116.4	26.2	1.00 (0.76–1.33)
Ischemic heart disease	339	2449.9	138.4	379	3,734.5	101.5	1.05 (0.90–1.22)
Stroke	512	2313.3	221.3	567	3,419.1	165.8	0.99 (0.88–1.12)
Transient ischemic attack	76	2737.3	27.8	92	4,137.2	22.2	0.97 (0.72–1.32)
Heart failure	641	2192.4	292.4	733	3,301.7	222.0	1.00 (0.90–1.11)
Coronary revascularization (treatment)	30	2770.9	10.8	43	4,198.5	10.2	0.85 (0.51–1.42)
All-cause death	2	1401.9	1.4	2	2,034.6	1.0	1.46 (0.20–10.68)

*All-cause death: includes two patients in each of the two groups

Coronary revascularization is a treatment code that has secondary outcome variables and overlapping values

**Table 5:** Risk of cardiovascular diseases in the two drug groups after matching between 2015 and 2018 (without cardiovascular disease history)

Classification	Febuxostat (24,952 patients)			Allopurinol (24,952 patients)			HR (95% CI)
	Number of patients (*n*)	Person–years	Incidence rate (1,000 person –years)	Number of patients (*n*)	Person–years	Incidence rate (1,000 person –years)	
*Primary outcome variable**							
Cardiovascular disease	531	22,964.2	23.1	772	37,606.7	20.5	1.11 (0.99–1.24)
*Secondary outcome variable*							
Myocardial infarction	51	23,349.7	2.2	75	38,356.3	2.0	1.09 (0.76–1.57)
Ischemic heart disease	145	23,289.0	6.2	227	38,192.3	5.9	1.02 (0.83–1.27)
Stroke	131	23,286.3	5.6	209	38,229.8	5.5	1.06 (0.85–1.33)
Transient ischemic attack	56	23,338.0	2.4	85	38,342.4	2.2	1.06 (0.75–1.49)
Heart failure	247	23,190.6	10.7	326	38,106.3	8.6	1.21 (1.02–1.44)
Coronary revascularization (treatment)	26	23,368.6	1.1	45	38,388.6	1.2	0.88 (0.52–1.49)
All-cause death	3	22,964.2	0.1	5	37,606.7	0.1	1.38 (0.31–6.23)

Note 1) * All-cause death: includes 3 and 5 patients in the febuxostat and allopurinol groups, respectively

Note 2) Coronary revascularization is a treatment code that has secondary outcome variables and overlapping values

**Table 6:** Risk of cardiovascular diseases in the two drug groups after matching between 2016 and 2018 (with cardiovascular disease history)

Classification	Febuxostat (5466 patients)			Allopurinol (5466 patients)			HR (95% CI)
	Number of patients (*n*)	Person–years	Incidence rate (1000 person –years)	Number of patients (*n*)	Person–years	Incidence rate (1000 person –years)	
*Primary outcome variable**							
Cardiovascular disease	2747	2566.6	1070.3	2769	2883.0	960.4	1.03 (0.98–1.09)
*Secondary outcome variable*							
Myocardial infarction	164	4,683.2	35.0	184	5,274.6	34.9	0.94 (0.76–1.16)
Ischemic heart disease	641	4,274.7	150.0	663	4,853.4	136.6	1.02 (0.91–1.13)
Stroke	1,016	3,977.9	255.4	1053	4,465.3	235.8	0.99 (0.91–1.08)
Transient ischemic attack	192	4,659.6	41.2	193	5,259.0	36.7	1.04 (0.85–1.27)
Heart failure	1,115	3,944.0	282.7	1176	4,389.1	267.9	0.99 (0.91–1.07)
Coronary revascularization (treatment)	51	4,775.3	10.7	50	5,399.2	9.3	1.09 (0.71–1.67)
All-cause death	4	2,566.6	1.6	4	2,883.0	1.4	1.16 (0.29–4.65)

*All-cause death: includes 4 patients in each of the two groups

Coronary revascularization is a treatment code that has secondary outcome variables and overlapping values

**Table 7:** Risk of cardiovascular diseases in the two drug groups after matching between 2016 and 2018 (without cardiovascular disease history)

Classification	Febuxostat (21,402 patients)			Allopurinol (21,402 patients)			HR (95% CI)
	Number of patients (*n*)	Person–years	Incidence rate (1000 person –years)	Number of patients (*n*)	Person–years	Incidence rate (1000 person –years)	
*Primary outcome variable**							
Cardiovascular disease	362	17,737.3	20.4	386	20,984.8	18.4	1.11 (0.96–1.28)
*Secondary outcome variable*							
Myocardial infarction	33	17,952.1	1.8	31	21,226.4	1.5	1.28 (0.78–2.09)
Ischemic heart disease	102	17,913.8	5.7	105	21,180.4	5.0	1.15 (0.87–1.50)
Stroke	90	17,913.7	5.0	114	21,168.2	5.4	0.93 (0.70–1.22)
Transient ischemic attack	41	17,939.0	2.3	38	21,218.1	1.8	1.28 (0.82–2.00)
Heart failure	170	17,854.6	9.5	165	21,140.3	7.8	1.22 (0.98–1.51)
Coronary revascularization (treatment)	18	17,959.0	1.0	16	21,236.4	0.8	1.46 (0.70–3.05)
All-cause death	2	17,737.3	0.1	3	20,984.8	0.1	0.87 (0.14–5.24)

*All-cause death: includes 2 and 3 patients in the febuxostat and allopurinol groups, respectively

Coronary revascularization is a treatment code that has secondary outcome variables and overlapping values

**Table 8:** Baseline characteristics before and after matching in the febuxostat and allopurinol groups between 2016 and 2018

Classification		Before propensity score matching					After propensity score matching				
		Febuxostat (29,412 patients, 43.1%)		Allopurinol (38,905 patients, 57.0%)		STD (%)	Febuxostat (26,962 patients, 50.0%)		Allopurinol (26,962 patients, 50.0%)		STD (%)
		N	(%)	N	(%)		N	(%)	N	(%)	
Sex	Men	25,856	87.9	34,524	88.7	− 2.6	24,067	89.3	24,302	90.1	− 2.9
Age	Mean (standard deviation)	49.3 (± 17.0)		48.1 (± 16.4)		4.2	48.1 (± 16.4)		47.6 (± 16.2)		3.1
Medication history	Nonsteroidal anti-inflammatory drugs (NSAIDs)	22,880	77.8	30,104	77.4	1.0	21,109	78.3	21,335	79.1	− 2.0
	Systemic corticosteroids	17,830	60.6	22,759	58.5	4.3	16,486	61.2	16,676	61.9	− 1.4
	Gout medication	13,535	46.0	19,089	49.1	− 6.1	13,020	48.3	13,288	49.3	− 2.0
	Anti-hyperlipidemic agents	9152	31.1	9013	23.2	17.9	7488	27.8	7265	27.0	1.9
	Hypoglycemic agents	3650	12.4	3492	9.0	11.1	2722	10.1	2628	9.8	1.2
	Hypotensors	10,717	36.4	11,179	28.7	16.5	8,702	32.3	8504	31.5	1.6
CCI	Mean (standard deviation)	1.3 (± 1.5)		1.0 (± 1.3)		21.4	1.1 (± 1.4)		1.1 (± 1.3)		0.0
	0	12,261	41.7	19,245	49.5	− 15.7	12,254	45.5	12,655	46.9	− 3.0
	1	6734	22.9	9660	24.8	− 4.5	6707	24.9	6757	25.1	− 0.4
	2	3577	12.2	4265	11.0	3.8	3270	12.1	3107	11.5	1.9
	3	2,076	7.1	2152	5.5	6.3	1697	6.3	1608	6.0	1.4
	> 3	4764	16.2	3583	9.2	21.1	3034	11.3	2835	10.5	2.4
Disease history	Renal failure	3912	13.3	1571	4.0	33.4	1585	5.9	1570	5.8	0.2
	Kidney stone	302	1.0	312	0.8	2.4	229	0.9	229	0.9	0.0
	Angina	2689	9.1	2563	6.6	9.5	2006	7.4	1921	7.1	1.2
	Diabetes mellitus	6623	22.5	6375	16.4	15.5	5162	19.2	4983	18.5	1.7
	Peripheral arterial disease	22	0.1	14	0.0	1.6	19	0.1	12	0.0	1.1
	Degenerative arthritis	3431	11.7	3675	9.5	7.2	3,123	11.6	3066	11.4	0.7
	Hypertension	12,641	43.0	13,232	34.0	18.5	10,349	38.4	10,087	37.4	2.0
	Hyperlipidemia	13,095	44.5	13,547	34.8	19.9	11,081	41.1	10,830	40.2	1.9
	Obesity	56	0.2	53	0.1	1.3	54	0.2	39	0.1	1.3
	Smoking	29	0.1	34	0.1	0.4	22	0.1	23	0.1	− 0.1
History of cardiovascular disease	Myocardial infarction	438	1.5	416	1.1	3.7	327	1.2	304	1.1	0.8
	Ischemic heart disease	1377	4.7	1287	3.3	7.0	1001	3.7	966	3.6	0.7
	Stroke	1668	5.7	1694	4.4	6.0	1266	4.7	1186	4.4	1.4
	Transient ischemic attack	332	1.1	386	1.0	1.3	264	1.0	263	1.0	0.0
	Heart failure	2268	7.7	1819	4.7	12.6	1,589	5.9	1,509	5.6	1.3
	Coronary revascularization	594	2.0	538	1.4	4.9	438	1.6	392	1.5	1.4

Medication history, CCI, disease history: results of use or diagnosis for 1 year prior to the index date

If the absolute value of the standardized difference (STD) was higher than 10, it was judged to be different between the two groups

Propensity score matching: propensity scores of the febuxostat and allopurinol groups were matched 1:1 with a caliper width of 0.005

Average follow-up of 305 days in the febuxostat and allopurinol groups between 2016 and 2018

**Table 9:** Definitions of outcomes (diagnoses, procedures, and treatments) based on International Classification of Diseases (ICD-10) codes

ICD-10 code						
Myocardial infarction	I21*	Acute myocardial infarction				
	I22*	Subsequent myocardial infarction				
	I23*	Certain current complications following acute myocardial infarction				
	I25.2	Old myocardial infarction				
Ischemic heart disease	I24*	Other acute ischemic heart diseases				
	I25* (I25.2 Excluded)	Chronic ischemic heart disease				
Stroke	I60*	Subarachnoid hemorrhage				
	I61*	Intracerebral hemorrhage				
	I62*	Other nontraumatic intracranial hemorrhage				
	I63*	Cerebral infarction				
	I64*	Stroke, not specified as hemorrhage or infarction				
Transient ischemic attack	G45*	Transient cerebral ischemic attacks and related syndromes				
Heart failure	I50*	Heart failure				
Death	I46.1	Sudden cardiac death, so described				
	R96*	Other sudden death, cause unknown				
	R98	Unattended death				
	R99	Other ill-defined and unspecified causes of mortality				
EDI code						
Coronary revascularization	M6551	Percutaneous coronary intervention (PCI)				
	M6552					
	M6561					
	M6563					
	M6564					
	M6571					
	M6572					
	O1641	Coronary artery bypass graft (CABG)				
	O1642					
	O1647					
	OA641					
	OA642					
	OA647					

*Include all subcodes

**Table 10:** Definitions of covariate drugs based on Anatomical Therapeutic Chemical (ATC) codes

ATC code			
Hypotensive agents	ACEI	Captopril	C09AA01, C09BA01
		Cilazapril	C09AA08
		Enalapril	C09AA02, C09BA02
		Fosinopril	C09AA09
		Imidapril	C09AA16
		Lisinopril	C09AA03, C09BA03
		Perindopril	C09AA04, C09AA04, C09BA04
		Quinapril	C09AA06
		Ramipril	C09AA05, C09BA05, C09BB05
		Temocapril	C09AA14
		Alacepril	C09AA
		Zofenopril	C09AA15
	CCB	Amlodipine	C08CA01, C09DB01, C09DB02, C09DB04, C09DB06, C09DB07, C09DX03, C10BX03, C10BX09, C09DX, C10BX
		Felodipine	C08CA02, C08CA02, C09BB05, C07FB02
		Isradipine	C08CA03
		Nicardipine	C08CA04, C08CA04
		Nifedipine	C08CA05
		Nimodipine	C08CA06
		Nitredipine	C08CA08, C09AA02
		Lacidipine	C08CA09
		Nilvadipine	C08CA10
		Manidipine	C08CA11
		Barnidipine	C08CA12
		Lercanidipine	C08CA13, C09DB08
		Cilnidipine	C08CA14
		Benidipine	C08CA15
		Verapamil	C08DA01
		Nisoldipine	C08CA07
		Diltiazem	C08DB01
	ARB	Losartan	C09CA01, C09DA01, C09DB06, C09DX, C10BX
		Eprosartan	C09CA02, C09DA02
		Valsartan	C09DX04, C09CA03, C09DA03, C10BX10, C09DB01, C09DB08
		Irbesartan	C09CA04, C09DA04, C10BX
		Candesartan	C09DB07, C10BX
		Telmisartan	C09DB04, C10BX, C09DX
		Olmesartan	C09DB02, C09DX03, C10BX
		Fimasartan	C09CA10
	Diuretics	Chlorthalidone	C03BA04, C07CB03
		Indapamide	C03BA11, C03BA11, C09BA04
		Hydrochlorothiazide	C03AA03, C07BB07, C09DX03, C09DX, C03EA01
Hypoglycemic agent	Sodium glucose contransporter-2 inhibitor	Dapagliflozin	A10BX09, A10BD15
		Empagliflozin	A10BX12, A10BD20
		Ipragliflozin	A10BX
	Dipeptidyl peptidase-4 inhibitor	Sitagliptin	A10BH01, A10BD07
		Saxagliptin	A10BH03, A10BD10
		Linagliptin	A10BH05, A10BD11
		Alogliptin	A10BH04, A10BD13, A10BD09
		Vildagliptin	A10BH02, A10BD08
		Gemigliptin	A10BH06, A10BH52, A10BD18
	Sulfonylureas	Glimepiride	A10BB12, A10BD02, A10BD04, A10BD06
		Glibenclamide	A10BB01, A10BB02, A10BD02
		Gliclazide	A10BB09
		Glipizide	A10BB07
		Gliquidone	A10BB08
		Tolazamide	A10BB05
		Chlorpropamide	A10BB02
	Meglitinides	Mitiglinide	A10BX08
		Nateglinide	A10BX03
		Repaglinide	A10BX02, A10BD14
	Thiazolidinedione	Pioglitazone	A10BG03, A10BD09, A10BD06, A10BD05
		Rosiglitazone	A10BG02, A10BD04, A10BD03
		Lobeglitazone	A10BG
	α-Glucosidase inhibitors	Acarbose	A10BF01
		Miglitol	A10BF02
		Voglibose	A10BF03, A10BD
	Glucagon-Like Peptide-1 Receptor Agonist	Albiglutide	A10BX13
		Dulaglutide	A10BX14
		Exenatide	A10BX04, A10BJ01
		Lixisenatide	A10BX10, A10BJ03
	Metformin		A10BA02, A10BH, A10BD, A10BD15, A10BD22, A10BD18, A10BD02, A10BD11, A10BD03, A10BX03, A10BD14, A10BD10, A10BD07, A10BD08, A10BH, A10BD05
Corticosteroid		Hydrocortisone	H02AB09
		Betamethasone	H02AB01
		Dexamethasone	H02AB02
		Methylprednisolone	H02AB04
		Prednisolone	H02AB06, H02AB04, S02BA03
		Triamcinolone	H02AB08
		Deflazacort	H02AB13
Antigout preparations		Probenecid	M04AB01
		Colchicine	M04AC01
Nonsteroidal anti-inflammatory drugs	NSAIDs	Aspirin	N02BA01, B01AC06, B01AC30, M03BA53, N02BA01, N02BA51, R06AA57
		Ethenzamide	N02BE51, R05FA02, R05X
		Salsalate	N02BA06
		Flufenamic Acid	M01AG03
		Mefenamic Acid	M01AG01
		Tolfenamic Acid	M01AG02
		Aceclofenac	M01AB16
		Felbinac	M02AA08
		Acemetacin	M01AB11
		Etodolac	M01AB08
		Indometacin	C01EB03, M01AB01
		Ketorolac	M01AB15
		Lonazolac	M01AB09
		Proglumetacin	M01AB14
		Sulindac	M01AB02
		Nabumetone	M01AX01
		Alminoprofen	M01AE16
		Dexibuprofen	M01AE14
		Dexketoprofen	M01AE17, N02AJ14
		Fenoprofen	M01AE04
		Flurbiprofen	M01AE09
		Ibuprofen	M01AE01, M01AE51, N02AJ09, R01BA52
		Ibuproxam	M01AE13
		Ketoprofen	M01AE03
		Naproxen	M01AE02, M01AE52
		Oxaprozin	M01AE12
		Pranoprofen	M01AE
		Tiaprofenic Acid	M01AE11
		Lornoxicam	M01AC05
		Meloxicam	M01AC01, M01AC06
		Piroxicam	M01AC01
		Tenoxicam	M01AC02
		Dichloralphenazone	N02CX
		Nimesulide	M01AX17
		Celecoxib	M01AH01
		Etoricoxib	M01AH05
		Benzydamine	M01AX
		Imidazole Salicylate	N02BA16
		Morniflumate	M01AX22
		Nefopam	N02BG06
Antihyperlipidemic agents	Statins	Atorvastatin	C10AA05, C10BX03, A10BD, C10BX, C10BA05
		Fluvastatin	C10AA04
		Lovastatin	C10AA02
		Pitavastatin	C10AA08, C10BX
		Pravastatin	C10AA03, C10BA03
		Rosuvastatin	C10AA07, C10BX, C10BX09, C10BA06, A10BH52, A10BD, C10BA07, C10BX10
		Simvastatin	C10AA01, C10BA02, C10BA04
	Fibrates	Gemfibrozil	C10AB04
		Fenofibrate	C10AB05, C10BA04, C10BA03
		Choline fenofibrate	C10AB11
		Bezafibrate	C10AB02
	Cholesterol absorption inhibitor	Ezetimibe	C10BA05, C10BA06, C10BA02
	Other lipid modifying agents	Omega-3-triglyceride	C10AX06, C10BA07
